# Augmented low-Dye tape alters foot mobility and neuromotor control of gait in individuals with and without exercise related leg pain

**DOI:** 10.1186/1757-1146-3-5

**Published:** 2010-03-18

**Authors:** Melinda Franettovich, Andrew R Chapman, Peter Blanch, Bill Vicenzino

**Affiliations:** 1 The University of Queensland, Brisbane, Australia; 2 The Australian Institute of Sport, Canberra, Australia; 3 McGill University, Montreal, Canada

## Abstract

**Background:**

Augmented low-Dye (ALD) tape is frequently used in the management of lower limb musculoskeletal pain and injury, yet our knowledge of its effect is incomplete, especially in regard to its neuromotor effects.

**Methods:**

We measured electromyographic (EMG) activity of twelve lower limb muscles, three-dimensional kinematics of the ankle, knee, hip and pelvis, foot posture and foot mobility to determine the physiological effect of ALD tape. Fourteen females with exercise related leg pain and 14 matched asymptomatic females walked on a treadmill under three conditions: pre-tape, tape and post-tape. A series of repeated measure analysis of variance procedures were performed to investigate differences in EMG, kinematic, foot posture and mobility measurements.

**Results:**

Application of ALD tape produced reductions in recruitment of tibialis anterior (7.3%) and tibialis posterior (6.9%). Large reductions in midfoot mobility (0.45 to 0.63 cm) and increases in arch height (0.58 cm), as well as moderate changes in ankle motion in the sagittal (2.0 to 5.3°) and transverse planes (4.0 to 4.3°) were observed. Reduced muscle activation (<3.0%) and increased motion (<1.7°) was observed at more proximal segments (knee, hip, pelvis) but were of smaller magnitude than at the foot and ankle. Changes in foot posture, foot mobility, ankle kinematics and leg muscle activity did not persist following the removal of ALD tape, but at more proximal segments small changes (<2.2°, <5.4% maximum) continued to be observed following the removal of tape. There were no differences between groups.

**Conclusions:**

This study provides evidence that ALD tape influences muscle recruitment, movement patterns, foot posture and foot mobility. These effects occur in individuals with and without pain, and are dissipated up the kinetic chain. ALD tape should be considered in the management of individuals where increased arch height, reduced foot mobility, reduced ankle abduction and plantar flexion or reduced activation of leg muscles is desired.

## Background

The augmented low-Dye (ALD) is a taping technique frequently used by clinicians in the management of lower limb musculoskeletal pain and injury. A recent review of the literature concluded that ALD tape produces a biomechanical effect, specifically by increasing medial longitudinal arch height, reducing calcaneal eversion and tibial internal rotation, reducing medial forefoot pressures and increasing lateral midfoot pressures during standing, walking and jogging [1]. The review also found preliminary evidence of a neuromuscular effect, specifically reduced tibialis posterior and tibialis anterior activation during walking [1,2]. In addition, the review highlighted that our current knowledge of its effects is incomplete. For example, investigations have been performed primarily in asymptomatic cohorts. Whilst these investigations remove pain as a confounder and allow researchers to make inferences about the mechanism of the intervention, ultimately these investigations must be replicated in a symptomatic cohort to be reflective of clinical practice. Secondly, we also do not understand the effect of ALD tape on lower limb movement patterns as previous biomechanical investigations have been limited to foot and leg posture and plantar pressure distribution. Finally, tape-induced reductions in pain have been reported to continue following the removal of tape [3], but there has been no such investigation of the biomechanical and neuromuscular effects.

The purpose of this study was to investigate the biomechanical (lower limb movement patterns, foot posture and foot mobility) and neuromuscular (muscle recruitment patterns) effects of ALD tape in individuals with and without exercise related leg pain (ERLP) while tape was in situ and immediately following its removal. We hypothesized a reduction in lower limb muscle activity and range of movement, regardless of symptomatic status, and that tape-induced effects would continue immediately following removal of the tape.

## Methods

### Participants

Fourteen females with a history of ERLP in the twelve months prior to the study were recruited. ERLP was defined as pain located between the ankle and the knee, which is experienced with weight bearing activities and ceases/diminishes when activity ceases [4,5]. The term includes clinical labels such as shin pain, shin splints, medial tibial stress syndrome and periostitis. Individuals did not have point bone tenderness on palpation of the posterior-medial border of the tibia, and for the purposes of this study, individuals were excluded if there was a medical diagnosis of compartment syndrome or tibial stress fracture. Participants were also excluded if there were signs and symptoms of radiculopathy or other neurological involvement, or if symptoms were provoked with walking (experimental activity) as we did not want to confound results with the direct concurrent effect of pain on muscle activity and motion. Fourteen age, weight and height matched asymptomatic control females were also recruited. These individuals did not have a lower limb injury in the twelve months prior to the study that interfered with work/leisure activities or required treatment. Individuals were excluded from either group if a history of surgery to the lower limb, blood clotting or bleeding abnormalities, a neurological or cardiac condition, or allergy to tape was reported. All individuals provided informed written consent and the study was approved by the institutional human research ethics committees.

### Procedure

**Figure 1:**
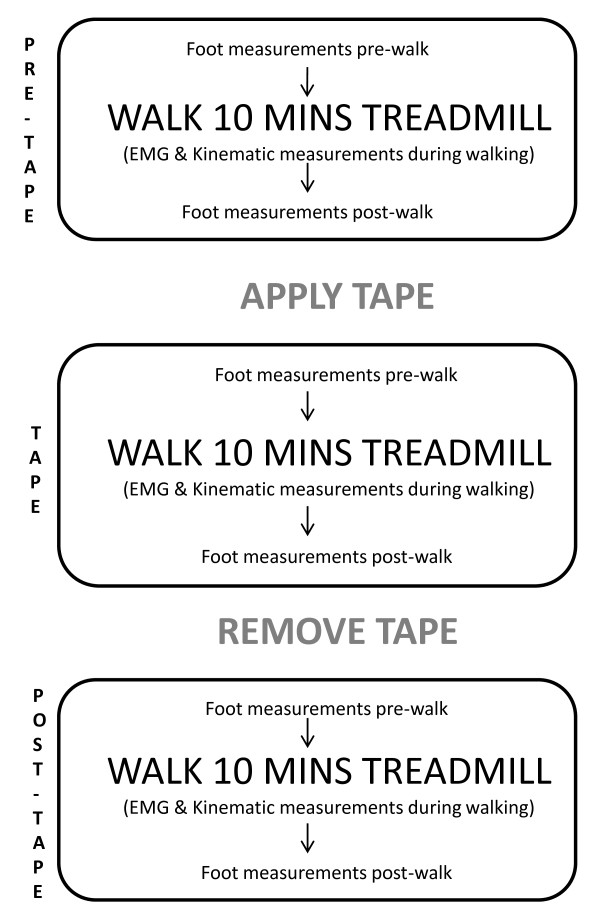
**Experimental procedure**.

Participants walked on a treadmill for ten minutes under three conditions: pre-tape, tape, post-tape (Figure 1). For each individual, walking speed was self-selected ("comfortable") and was standardized between conditions. Running was not assessed because it was a pain provocative activity for some individuals in the ERLP group and we did not want to confound results with the direct concurrent effect of pain on muscle activity and motion. Electromyographic (EMG) and kinematic data were recorded during the ten minutes of walking and foot posture and mobility data were measured before (pre) and after walking (post) for all three conditions.

### ALD tape

ALD tape was applied by the same physiotherapist and has been described previously [1,2,6]. It comprises the low-Dye technique (spurs and mini-stirrups) plus three reverse sixes and two calcaneal slings anchored to the lower third of the leg. The tape is applied with the talocrural joint in plantigrade and the rearfoot in two-thirds supination. A rigid sports tape (38 mm zinc oxide adhesive, Leukosport BDF) was used.

### EMG

We measured EMG activity (Noraxon Telemyo) from tibialis posterior (TP), tibialis anterior (TA), peroneus longus (PL), medial and lateral gastrocnemius (MG, LG), soleus (SOL), vastus medialis obliquus (VMO), vastus lateralis (VL), rectus femoris (RF), semitendinosus (ST), biceps femoris (BF) and gluteus medius (GM). Bipolar silver/silver chloride surface electrodes (10 mm diameter contact area, 20 mm fixed inter-electrode distance, Nicolet Biomedical) were used for recordings from all muscles except TP. An intramuscular recording was chosen for TP due to its deep location to reduce contamination from attenuation of signal or crosstalk from overlying muscles [2,7]. Bipolar intramuscular electrodes were fabricated from two strands of Teflon^® ^coated stainless steel wire (California Wire Company) that were inserted into a hypodermic needle (0.41 × 32 mm). 2 mm of Teflon coating was removed from the end of each wire and to prevent contact the exposed tips were bent back by 2 mm and 4 mm. Intramuscular electrodes were inserted with the guidance of real-time ultrasound (Toshiba Nemio 20) using an established procedure [8,9]. The application of all electrodes followed established standards in the literature [10-12]. Electrodes were positioned according to published recommendations based on innervation zone locations [10-12]. EMG data was sampled at 3000 Hz and band-pass filtered between 10 and 1000 Hz.

### Kinematics

Three dimensional motion analyses of the ankle, knee, hip and pelvis was performed using an eight camera VICON system (Oxford Metrics, UK) sampling at 250 Hz. Retroflective markers were placed according to the Plug In Gait^® ^model (Oxford Metrics, UK) which was used for determination of kinematic data [13,14]. Joint rotations were referenced to standing position. Ankle motion was not derived in the frontal plane because only two markers defined the foot segment [14].

### Foot posture and foot mobility

A purpose-built platform was used to perform all foot posture and mobility measurements, as previously described [15]. Measurements of foot posture (weight bearing and non-weight bearing arch height and midfoot width) were used to calculate three indices of foot mobility. Differences between non-weight bearing and weight bearing measurements of arch height and midfoot width (termed arch height difference, midfoot width difference) were calculated as indices of the vertical and medio-lateral motion of the midfoot, respectively [15]. A composite measure of vertical and medio-lateral motion of the midfoot, foot mobility magnitude, was based on Pythagorean theorem and calculated with the formula: Foot mobility magnitude = √((difference in arch height)^2 ^+ (difference in midfoot width)^2^) [15].

### Data management

Signal processing procedures were consistent for all individuals and all three conditions. EMG data was adjusted for DC offset, full-wave rectified and filtered with a 4^th ^order high-pass Butterworth filter with a 10 Hz cutoff. TP and SOL recordings contained increased signal artifact and high-pass cutoffs of 50 Hz for TP and 20 Hz for SOL were used in place of 10 Hz [16,17]. EMG data was amplitude normalised to the maximum amplitude of activity from the pre-tape condition [2,18]. For kinematic data a generalising cross validatory spline was used to remove low frequency artefact from marker trajectories[19].

Ten consecutive strides (foot contact to ipsilateral foot contact) from each minute of data were selected for analysis [20]. Kinematic and EMG data were time normalized to 100 points for each stride and data were averaged across the ten minutes for each condition (i.e. ten strides per ten minutes of data = average of 100 strides per condition).

### Data analysis

Amplitude (peak, stance phase average, swing phase average) and temporal (time to peak, duration, onset and offset of activity) characteristics of muscle activity were calculated from EMG recordings to provide a comprehensive description of muscle recruitment patterns i.e. amount of activation as well as timing of activation [2]. Minimum, maximum and total excursion in each plane at the ankle, knee, hip and pelvis was derived from kinematic data.

A series of two-way repeated measure analysis of variance (ANOVA) procedures (SPSS 16.0 for Windows) with between subjects factor of GROUP (control and ERLP) and within subject factor of TIME (pre-tape, tape, post-tape) were performed to investigate differences in EMG, kinematic, foot posture and foot mobility measurements (p < 0.05). Significant effects on ANOVA were followed up with tests of simple effects for pairwise comparisons between pre-tape and tape and between pre-tape and post tape (Bonferonni corrected p < 0.025). To provide an estimate of the treatment effect and as a proxy for an estimate of the clinical meaningfulness of the effect, standardised mean differences (SMD = mean difference/pooled standard deviation) were calculated. SMD greater than 1.2 were considered large, 0.6 to 1.2 moderate and less than 0.6 were considered small [21]. On the basis of a previous pilot study [2] we anticipated a large effect of tape. Power calculations indicated 14 subjects per group would be adequate to detect such effects (SMD >1.2) at a power of 80% and ***p ***value of 0.05 [22]. Results are presented as mean difference (95% confidence interval).

## Results

As Table 1 demonstrates, participants were evenly matched for age, weight and height. Participants in the ERLP group reported mild pain (mean: 14.3 mm (1-49 mm) on visual analogue scale), which was on average 32.5 months in duration (2-32 months). The mean duration since symptoms were last experienced was 3.6 weeks (range: 0-12 weeks).

**Table 1:** Participant characteristics

	Asymptomatic control Mean (SD)	ERLP Mean (SD)	*p*-value
Age (yrs)	25.5 (6.2)	25.9 (5.5)	0.85
Weight (kg)	63.5 (6.8)	62.2 (6.1)	0.86
Height (cm)	166.4 (6.6)	166.0 (5.2)	0.60
Duration of symptoms (months)	N/A	32.5 (36.1)	N/A
Duration since last symptoms (weeks)	N/A	3.6 (5.5)	N/A
Pain Visual Analogue Scale (100 mm)	N/A	14.3 (12.7)	N/A

The repeated measures ANOVA (for detail see additional file 1) revealed that there was a statistically significant effect of TIME (*p *< 0.05) for all measurements of foot posture, foot mobility, motion at all lower limb joints in each plane, and activation of all muscles except for GM and SOL. There was no GROUP by TIME interaction effect for all variables except PL average stance phase activity (*p *= 0.049), MG duration of activity (*p *= 0.046), and ST onset of activity (*p *= 0.010). This indicates that for the majority of EMG, kinematic and foot posture/mobility data, the effect of tape (TIME main effect) was not significantly different between individuals with and without ERLP (GROUP main effect). It was therefore decided to pool data from these groups in follow up tests of simple effects for TIME for all variables except PL average stance phase activity (there was not a significant TIME effect for MG duration of activity (*p *= 0.12) or ST onset of activity (*p *= 0.10)). The results of follow up tests of simple effects for TIME on the pooled data (n = 28) are presented in additional files 2, 3 and 4.

### The effect of ALD tape on lower limb muscle activity

**Figure 2:**
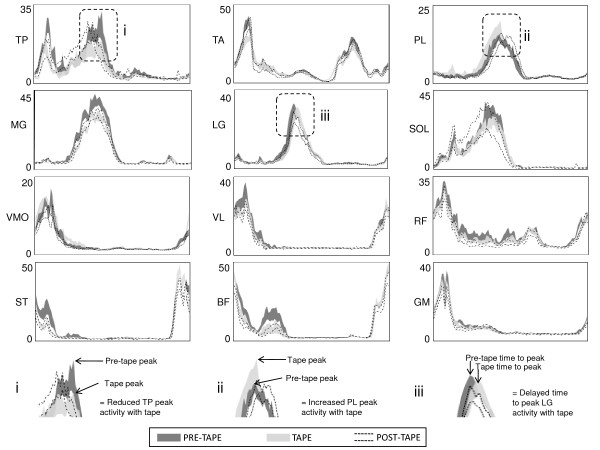
**Effect of ALD tape on lower limb muscle activity**. The 95% confidence interval of the mean muscle recruitment patterns for the pre-tape, tape and post-tape conditions for a representative individual. X-axis is 0-100% stride cycle; Y-axis is normalised EMG amplitude (% maximum). Panels i, ii, iii provide an example of interpretation of changes in muscle recruitment patterns that are described in the text.

A snapshot pictorial representation of the data is shown in Figure 2. With the application of tape stance phase amplitude of activity was reduced for TP [average: -1.6% maximum (95% CI: -2.9 to -0.3)], TA [peak: -7.3% maximum (95% CI: -0.7 to -4.8), average: -0.7% maximum (95% CI: -1.2 to -0.2)] and MG [peak: -3.0% maximum (95% CI: -5.4 to -0.6), average: -0.9% maximum (95% CI: -1.4 to -0.3)]. Peak and average amplitude of activity during swing phase was also reduced for TA [peak: -2.7% maximum (95% CI: -4.1 to -1.7) average: -0.9% maximum (95% CI: -1.4 to -0.5)]. For PL, an increase in average stance phase average activation by 1.0% maximum (95% CI: 0.3 to 1.7) was observed in the ERLP group. These changes were all small (SMD < 0.6) except for peak TA activity in stance phase, which was a moderate reduction (SMD = 0.9). Tape also produced small reductions (ranging from -2.0 to -0.3% maximum, SMD < 0.6) in amplitude of more proximal muscles such as VL, RF, and BF during stance phase and an increase in ST activity during swing phase (2.5% maximum, SMD = 0.2). Reductions in leg muscle activity were not maintained following the removal of tape. In contrast, for the thigh muscles small reductions in activity (-5.4 to -0.2% maximum, SMD < 0.6) continued to be observed following the removal of tape.

Application of tape delayed the time to peak activity for MG by 1.3% of the stride (95% CI: 0.7 to 2.0) and for LG by 0.8% (95% CI: 0.3 to 1.2). These changes equate to delays of 13.5, 8.3 and 6.2 ms respectively. SMDs indicate that these changes were small to moderate (SMD = 0.4 to 0.8). For the thigh muscles, time to peak activity occurred earlier in stance phase for BF [-1.4% (95% CI: -2.5 to -0.3)], earlier in swing phase for RF [-2.9% (95% CI: -4.4 to -1.5)] and was delayed by 2.0% stride (95% CI: 0.4 to 3.6) in stance phase for RF. These changes equate to 14.6, 31.2, 20.8 ms and SMDs indicate these changes were small (SMD < 0.3). Other temporal aspects (onset, offset, duration) were not different with the application of tape. The changes in timing of peak activity were not maintained following the removal of tape.

### The effect of ALD tape on lower limb motion

**Figure 3:**
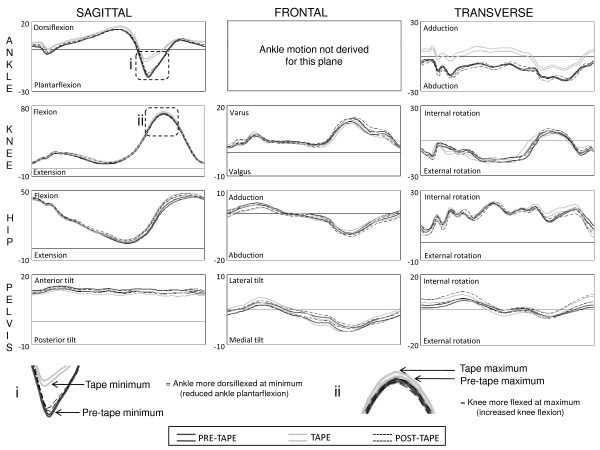
**Effect of ALD tape on lower limb motion**. The 95% confidence interval of the mean movement patterns for pre-tape, tape and post-tape conditions for a representative individual. X-axis is 0-100% stride cycle; Y-axis is degrees of movement. Panels i and ii provide an example of interpretation of changes in movement patterns that are described in the text.

Figure 3 illustrates movement patterns for the three conditions. With application of tape the ankle was more dorsiflexed and adducted at minimum [5.3° (95% CI: 3.9 to 6.7°) and 4.3° (95% CI: 3.0 to 5.6°), respectively] and maximum [2.0° (95% CI: 1.7 to 2.4°) and 4.1° (95% CI: 2.5 to 5.6°), respectively] excursions in the sagittal and transverse planes. Total sagittal plane motion was reduced [-3.1° (95% CI: -4.3 to -2.0°)]. These effects were moderate with SMDs of 0.5 to 1.1. Minimal changes were observed at the knee with small (SMD < 0.4) increases of 1.4° (95% CI: 0.8 to 2.0°) in knee flexion, 1.7° (95% CI: 0.8 to 2.5°) total sagittal plane excursion and 0.7° (95% CI: 0.1 to 1.4°) total frontal plane excursion. For the hip, small (SMD < 0.3) but significant changes ranging 0.7° to 2.1° were observed in the sagittal and transverse plane with increased total excursions due to increased hip flexion, internal and external rotation excursions. Application of tape produced a moderate (SMD = 1.0) increase in total excursion of the pelvis in the sagittal plane of 0.7° (95% CI: 0.5 to 0.8°) due to a more posterior tilted pelvic position. There were also small (SMD < 0.2) increases in total frontal and transverse plane excursion of the pelvis of 0.3° (95% CI: 0.1 to 0.6°) and 0.6° (95% CI: 0.1 to 1.1°).

Following removal of tape, ankle motion in the sagittal plane was not different to the pre-tape condition, but for the transverse plane there was increased ankle abduction [-0.7° (95% CI: -1.4 to -0.1°)], adduction [1.0° (95% CI: 0.3 to 1.7°)] and total excursion [1.7° (95% CI: 1.1 to 2.2°)]. However, these effects were small (SMD < 0.3). Tape induced changes at the knee in the sagittal plane continued to be observed following tape removal (ranging 0.5° to 1.4°), and increases in external rotation, internal rotation and total excursion in the transverse plane were also observed (ranging 1.0° to 2.2°). Again all changes were small in magnitude (SMD < 0.4). Similarly, tape induced changes in the sagittal and transverse planes at the hip were observed following removal of tape, as well as increased frontal plane movement, but all changes were small in magnitude (ranging 0.4° to 2.0°, SMD < 0.3). Following tape removal, the pelvis maintained a more posterior tilted position with a moderate (SMD = 0.9) increase total sagittal excursion of 0.6° (95% CI: 0.4 to 0.7°), and small (SMD < 0.4) increases in frontal and transverse plane excursion of 0.4° (95% CI: 0.1 to 0.7°) and 1.3° (95% CI: 0.8 to 1.7°).

### The effect of ALD tape on foot posture and mobility

**Figure 4:**
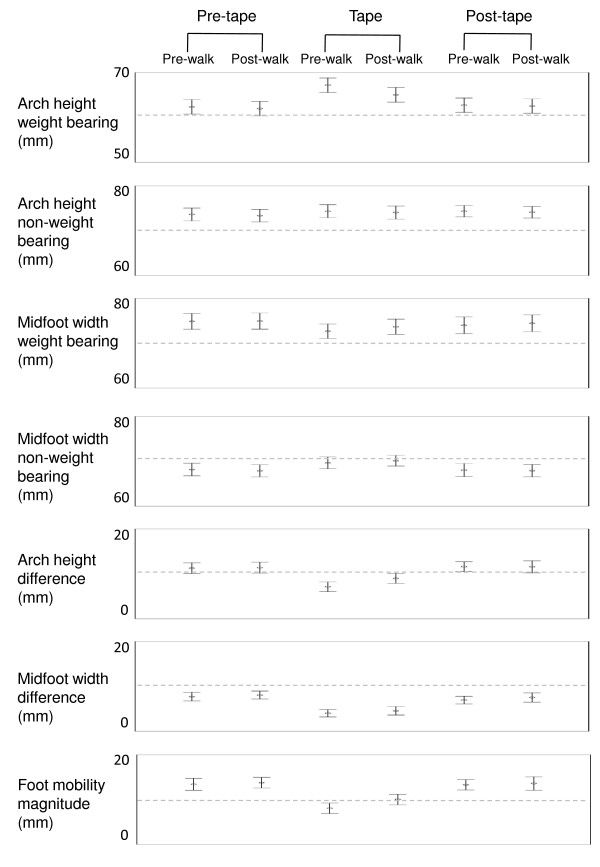
**Effect of ALD tape on foot posture and mobility**. The mean and 95% confidence interval for measurements of foot posture and mobility. X-axis is TIME (pre-tape, tape, post-tape); Y-axis is millimetres. Note that lower value is indicative of less mobility for arch height difference, midfoot width difference and foot mobility magnitude.

Figure 4 illustrates the effect of tape on foot posture and foot mobility. Application of tape produced a large (SMD = 1.3) increase in weight bearing arch height of 0.58 cm (95% CI: 0.54 to 0.62 cm) as well as large (SMD 1.4, 1.8, 1.9) reductions in arch height difference [-0.47 cm (95% CI: -0.54 to -0.40 cm)], midfoot width difference [-0.45 cm (95% CI: -0.52 to -0.38 cm)] and foot mobility magnitude [-0.63 cm (95% CI: -0.70 to -0.57 cm)]. Statistically significant changes were also observed for weight bearing midfoot width and non-weight bearing midfoot width and arch height but these changes were small (< 0.25 cm, SMD < 0.5). These effects were maintained following ten minutes of walking.

Immediately following removal of tape, there were some statistically significant differences in foot posture when compared to the pre-tape condition: weight bearing and non-weight bearing arch height remained increased by 0.09 cm (95% CI: 0.03 to 0.14 cm) and 0.11 cm (95% CI: 0.05 to 0.17 cm) respectively, and weight bearing midfoot width was reduced [-0.10 cm (95% CI: -0.16 to -0.05 cm]. However, the magnitudes of these effects were trivial (SMD < 0.2). Similarly, midfoot width difference remained reduced by 0.12 cm (95% CI: 0.04 to 0.20 cm) compared to the pre-tape condition, but this effect was small (SMD 0.5).

## Discussion

A substantive finding of this study was the similarity of the effect of ALD tape on foot mechanics and neuromotor control of gait (muscle recruitment and movement patterns) between injured and non-injured groups. This is an interesting finding because it appears to indicate the robustness of ALD-induced effects regardless of symptom status. It may also support the extrapolation of studies of ALD tape in asymptomatic individuals to those with ERLP.

Regardless of symptom status, we observed a moderate reduction in activation of TP and TA, a small reduction in MG activation, and a small increase in PL activation with application of ALD tape. This supports preliminary findings of tape-induced reductions in TP and TA activation in a small cohort (n = 5) of asymptomatic individuals [2]. We did not observe broad support for tape induced changes in temporal characteristics of muscle activity (i.e. onset, offset and duration of muscle activity) as expected from a preliminary trial [2], reinforcing reductions in activation levels as the primary neuromuscular effects. Although the underlying pathology of ERLP is not established, one hypothesis suggests that during stance the contraction of the superficial and deep flexors of the leg (TP, MG, LG, SOL, flexor digitorum longus, flexor hallucis longus), to control pronatory motions of the foot, exerts tension on the tibial fascia at its insertion onto the medial tibial crest [23]. The repetitive traction force that may occur with activity such as walking may result in injury to these soft tissues, the tibial fascia and/or its insertion into the medial tibial crest. In our study we observed tape induced reductions in activation of TP and MG. It is plausible that in reducing activity of TP and MG, tape may assist the resolution of symptoms and restoration of function by unloading symptomatic structures, thereby providing a possible mechanism underlying clinical efficacy of ALD in ERLP.

Large changes in sagittal and transverse plane motion at the ankle were observed with the application of tape. We found no previous report of the effect of ALD tape on three-dimensional lower limb motion, however, other studies may assist in the interpretation of our findings. For example, one mechanism through which ALD tape may help relieve ERLP is by reducing ankle abduction, since increased ankle abduction excursion (1.5°) during running was identified as a risk factor for development of ERLP [4] and in our study we observed that ALD tape reduced ankle abduction excursion by 4.3°. Although our observations were during walking, it appears that ALD tape may also be a useful technique for controlling ankle motion in running, and warrants further investigation of ALD tape as an intervention in this context.

ALD tape produced a large increase in arch height and large reductions in vertical and medio-lateral midfoot mobility through ten minutes of walking but not following removal of tape. These findings are novel and may underpin the reduction in muscle activity of two major foot-ankle muscles (TP, TA). This arguably supports the use of ALD tape in the management of individuals for whom it is clinically reasoned there exists a symptom related excessive motion of the foot. Controlling excessive motion and limiting deformation of soft-tissues may reduce tissue irritation and inflammation as proposed in the tissue stress model [24].

Apart from the local effects of ALD tape at the leg-ankle-foot segment there appears to be more broadly distributed effects seen by small reductions in activation of thigh muscles (VL, RF, ST, BF) and small changes in motion at the knee, hip and pelvic regions. Nevertheless, these changes at a distance from the taped region were larger than measurement error and should not be discounted, especially since in contrast to the local effects they remained after the removal of tape. It is difficult to speculate whether the distributed effects and their persistence following removal of tape are beneficial, harmful or inconsequential in the management of ERLP, but they may provide impetus for further enquiry in this regard.

A limitation of the current study is that we assessed lower limb muscle activity and motion during walking and yet ERLP is often related to more vigorous activities such as running. However, the reason we chose walking was because in this cohort running provoked the symptoms of several individuals and we felt it was important not to confound the results with the direct concurrent effect of pain on muscle activity and motion.

## Conclusions

ALD tape influences foot mobility and neuromotor control of gait regardless of the presence of ERLP. These effects are greatest at the foot and ankle and whilst the tape is in situ. Tape induced changes in neuromotor control of gait are dissipated up the kinetic chain, and in contrast to effects at the foot and ankle, changes in neuromotor control of proximal joints such as the knee, hip and pelvis continue to be observed following the removal of tape. The findings of the current study support the use of ALD tape in the management of individuals for whom increased arch height, reduced midfoot mobility, reduced ankle abduction and plantarflexion and/or reduced activity of the leg muscles is desired.

## List of abbreviations

ALD: Augmented low-Dye; BF: Biceps femoris; EMG: Electromyography; ERLP: Exercise related leg pain; GM: Gluteus medius; LG: Lateral gastrocnemius; MG: Medial gastrocnemius; PL: Peroneus longus; RF: Rectus femoris; SMD: Standardised mean difference; SOL: Soleus; ST: Semitendinosus; TA: Tibialis anterior; TP: Tibialis posterior; VL: Vastus lateralis; VMO: Vastus medialis obliquus

## Competing interests

The authors declare that they have no competing interests.

## Authors' contributions

MF contributed to conception and design, carried out acquisition of data, performed analysis and interpretation of data and drafted the manuscript. ARC contributed to conception and design, assisted with analysis and interpretation of data and assisted with revision of the manuscript. PB contributed to conception and design, assisted with analysis and interpretation of data and assisted with revision of the manuscript. BV contributed to conception and design, assisted with analysis and interpretation of data and assisted with revision of the manuscript.

## Supplementary Material

Additional file 1 **ANOVA statistics**. *p *values from GROUP by TIME repeated measure ANOVA. Click here for file

Additional file 2 **Effect of ALD tape on lower limb muscle activity**. Output from follow-up tests for TIME. Data based on pooled data from ERLP and control participants (n = 28). Click here for file

Additional file 3 **Effect of ALD tape on lower limb motion**. Output from follow-up tests for TIME. Data based on pooled data from ERLP and control participants (n = 28). Click here for file

Additional file 4 **Effect of ALD tape on foot posture and mobility**. Output from follow-up tests for TIME. Data based on pooled data from ERLP and control participants (n = 28). Click here for file

## References

[B1] Franettovich M Chapman A Blanch P Vicenzino B A physiological and psychological basis for anti-pronation taping from a critical review of the literature Sports Med 2008 38 617 631 10.2165/00007256-200838080-00001 18620463

[B2] Franettovich M Chapman A Vicenzino B Tape that increases medial longitudinal arch height also reduces leg muscle activity: a preliminary study Med Sci Sports Ex 2008 40 593 600 10.1249/MSS.0b013e318162134f 18317390

[B3] Saxelby J Betts RP Bygrave CJ "Low-dye" taping on the foot in the management of plantar-fasciitis Foot 1997 7 205 209 10.1016/S0958-2592(97)90037-7

[B4] Willems TM De Clercq D Delbaere K Vanderstraeten G De Cock A Witvrouw E A prospective study of gait related risk factors for exercise-related lower leg pain Gait Posture 2006 23 91 98 10.1016/j.gaitpost.2004.12.004 16311200

[B5] Reinking MF Hayes AM Intrinsic factors associated with exercise-related leg pain in collegiate cross-country runners Clin J Sport Med 2006 16 10 14 10.1097/01.jsm.0000188041.04760.d2 16377969

[B6] Vicenzino B Foot orthotics in the treatment of lower limb conditions: a musculoskeletal physiotherapy perspective Man Ther 2004 185 196 10.1016/j.math.2004.08.003 15522643

[B7] Perry J Easterday CS Antonelli DJ Surface versus intramuscular electrodes for electromyography of superficial and deep muscles Phys Ther 1981 61 7 15 7454803 10.1093/ptj/61.1.7

[B8] Chapman AR Vicenzino B Blanch P Knox JJ Hodges PW Leg muscle recruitment in highly trained cyclists J Sports Sci 2006 47 115 124 10.1080/02640410500131159 16368620

[B9] Hodges PW Kippers V Richardson CA Validation of a technique for accurate fine-wire electrode placement into posterior gluteus medius using real-time ultrasound guidance Electromyogr Clin Neurophysiol 1997 37 39 47 9063661

[B10] Hermens HJ Freriks B Disselhorst-Klug C Rau G Development of recommendations for SEMG sensors and sensor placement procedures J Electromyogr Kinesiol 2000 10 361 374 10.1016/S1050-6411(00)00027-4 11018445

[B11] Rainoldi A Melchiorri G Caruso I A method for positioning electrodes during surface EMG recordings in lower limb muscles J Neurosci Methods 2004 134 37 43 10.1016/j.jneumeth.2003.10.014 15102501

[B12] Perotto AO Anatomical guide for the electromyographer: the limbs and trunk 1994 3 Springfield, Illinois, USA: Charles C. Thomas

[B13] Growney E Meglan D Johnson M Cahalan T An K Repeated measures of adult normal walking using a video tracking system Gait Posture 1997 6 147 162 10.1016/S0966-6362(97)01114-4

[B14] Kadaba MP Ramakrishnan HK Wootten ME Measurement of lower extremity kinematics during level walking J Orthop Res 1990 8 383 392 10.1002/jor.1100080310 2324857

[B15] McPoil T Vicenzino B Cornwall M Collins N Warren M Reliability and normative values for the foot mobility magnitude: a composite measure of vertical and medio-lateral mobility of the midfoot J Foot Ankle 2009 2 6 10.1186/1757-1146-2-6 PMC2656480 19267907

[B16] Chapman AR Vicenzino B Blanch P Hodges PW Patterns of leg muscle recruitment vary between novice and highly trained cyclists J Electromyogr Kinesiol 2007 18 359 371 10.1016/j.jelekin.2005.12.007 17258470

[B17] Chapman AR Vicenzino B Blanch P Hodges PW Leg muscle recruitment during cycling is less developed in triathletes than cyclists despite matched cycling training loads Exp Brain Res 2007 181 503 518 10.1007/s00221-007-0949-5 17549464

[B18] Chapman AR Vicenzino B Blanch P Knox JJ Hodges PW Intramuscular fine-wire electromyography during cycling: repeatability, normalisation and a comparison to surface electromyography J Electromyogr Kinesiol 2010 20 108 117 10.1016/j.jelekin.2008.11.013 19339199

[B19] Woltring HJ A FORTRAN package for generalized, cross-validatory spline smoothing and differentiation Adv Eng Softw 1986 8 104 113

[B20] Zeni JA Richards JG Higginson JS Two simple methods for determining gait events during treadmill and overground walking using kinematic data Gait Posture 2007 27 710 714 10.1016/j.gaitpost.2007.07.007 17723303 PMC2384115

[B21] Hopkins WG Marshall SW Batterham AM Hanin J Progressive statistics for studies in sports medicine and exercise science Med Sci Sports Ex 2009 41 3 12 10.1249/MSS.0b013e31818cb278 19092709

[B22] Faul F Erdfelder E Lang A-G Buchner A G*Power 3: A flexible statistical power analysis program for the social, behavioural and biomedical sciences Beh Res Meth 2007 29 175 191 10.3758/bf03193146 17695343

[B23] Bouche RT Johnson CH Medial tibial stress syndrom (tibial fasciitis): A proposed pathomechanical model involving fascial traction J Am Podiatr Med Assoc 2007 97 31 36 17218623 10.7547/0970031

[B24] McPoil TG Hunt GC Evaluation and Management of Foot and Ankle Disorders: Present Problems and Future Directions J Orthop Sports Phys Ther 1995 21 381 388 7655482 10.2519/jospt.1995.21.6.381

